# Low Molecular Weight Protein Tyrosine Phosphatase Isoforms Regulate Breast Cancer Cells Migration through a RhoA Dependent Mechanism

**DOI:** 10.1371/journal.pone.0076307

**Published:** 2013-09-27

**Authors:** Irina Alho, Luis Costa, Manuel Bicho, Constança Coelho

**Affiliations:** 1 Genetics Laboratory, Cardiology Center, Faculdade de Medicina de Lisboa, Lisbon, Portugal; 2 Instituto de Medicina Molecular, Faculdade de Medicina de Lisboa, Lisbon, Portugal; 3 Serviço de Oncologia Médica, Departamento de Oncologia, Hospital de Santa Maria, Centro Hospital Lisboa Norte, Lisbon, Portugal; University of Birmingham, United Kingdom

## Abstract

Low molecular weight protein tyrosine phosphatase (LMW-PTP) has been associated with cell proliferation control through dephosphorylation and inactivation of growth factor receptors such as PDGF-R and EphA2, and with cellular adhesion and migration through p190RhoGap and RhoA. We aim to clarify the role of two main LMW-PTP isoforms in breast cancer tumorigenesis. We used a siRNA-mediated loss-of-function in MDA-MB-435 breast cancer cell line to study the role of the two main LMW-PTP isoforms, *fast* and *slow*, in breast cancer tumorigenesis and migration. Our results show that the siRNAs directed against total LMW-PTP and LMW-PTP *slow* isoform enhanced cell motility in an invasive breast cancer cell line, MDA-MB-435, with no changes in the proliferation and invasive potential of cells. The total LMW-PTP knockdown caused a more pronounced increase of cell migration. Suppression of total LMW-PTP decreased RhoA activation and suppression of the LMW-PTP *slow* isoform caused a small but significant increase in RhoA activation. We propose that the increase or decrease in RhoA activation induces changes in stress fibers formation and consequently alter the adhesive and migratory potential of cells. These findings suggest that the two main isoforms of LMW-PTP may act differentially, with the *fast* isoform having a more prominent role in tumor cell migration. In addition, our results highlight functional specificity among LMW-PTP isoforms, suggesting hitherto unknown roles for these proteins in breast cancer biology. Novel therapeutic approaches targeting LMW-PTP, considering the expression of these two isoforms and not LMW-PTP as a whole, should be investigated.

## Introduction

Protein tyrosine phosphatases (PTPs) and protein tyrosine kinases (PTKs) regulate the reversible phosphorylation of tyrosine residues in proteins, thus controlling vital physiological processes [1].

In humans, class II cysteine-based PTPs are represented by members of the Low Molecular Weight Protein Tyrosine Phosphatase (LMW-PTP) family, which are widely expressed, with no particular tissue specificity. LMW-PTP is encoded by the ACP1 (acid phosphatase locus 1) gene, located at 2p25, spanning 7 exons and 6 introns. The enzyme has two main isoforms, IF1 (*fast*) and IF2 (*slow*), both small enzymes consisting of only 157 amino acid residues and with a molecular weight of 18kDa. The amino acid sequence shows that the two isoforms arise from alternative and mutually exclusive splicing of exon 3 or 4 [2].

The 2 isoforms may have different roles in the progression of oncologic pathology [2,3]: the *fast* isoform is involved on migration, invasion and cell adhesion, catalysing the transformation of different substrates after platelet derived growth factor receptor (PDGF-R) stimulation, whilst the *slow* isoform, acting directly on PDGF-R, has growth factor receptors as substrates, *eg* platelet derived growth factor receptor (PDGF), leading to a decrease of cellular growth *via* its dephosphorylation [3]. LMW-PTP has been largely considered a negative regulator of growth factor-induced cell proliferation, although in some instances it acts as a positive regulator. Some proteins, such as Ephrin Receptor A2 (EphA2), seem to be involved in the regulation of carcinogenesis by LMW-PTP. Eph receptors are a family of receptor tyrosine kinases that have been shown to be overexpressed in a large number of cancers [4,5]. It is known that LMW-PTP has the potential to dephosphorylate EphA2 rendering it negatively regulated, which can increase transformation of normal epithelial cells, regulate tumor cell growth, survival, migration and invasion [1]. p190RhoGAP, a protein involved in the regulation of cytoskeleton rearrangement, is also dephosphorylated by LMW-PTP, with the consequent effect on RhoA [3]. This observation correlates with the influence of LMW-PTP expression on phenomena such as cell adhesion, spreading and migration [3].

Fang et al defend that enhanced RhoA activity is apparently regulated by enhanced LMW-PTP phosphatase activity and inhibition of tyrosine phosphorylation of p190RhoGAP, ultimately leading to the destabilization of cell-cell adhesion. They describe that overexpression of EphA2 promotes destabilization of adherens junctions through the axis LMW-PTP – p190RhoGAP – RhoA [6].

**Figure 1:**
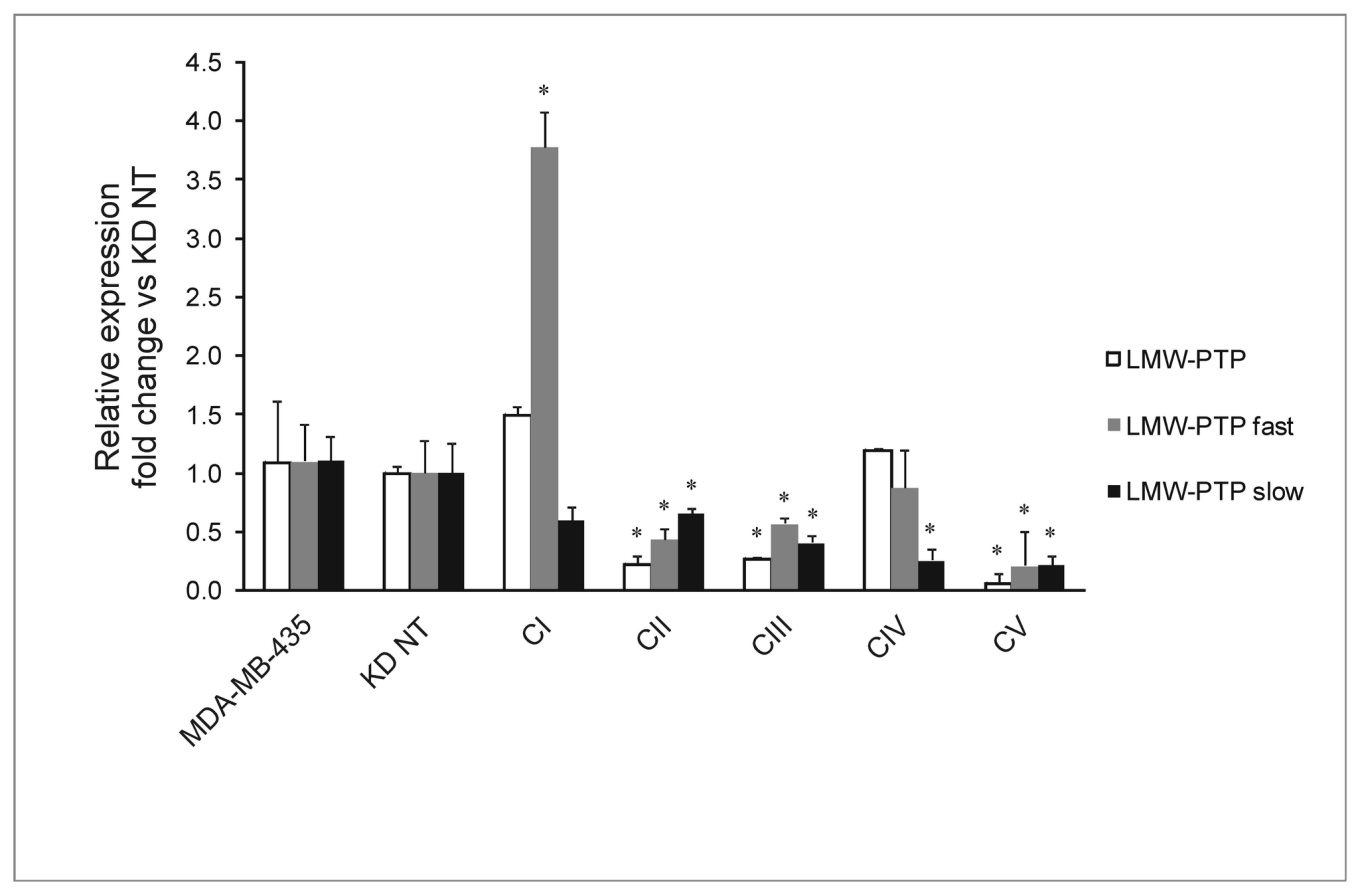
Relative expression of LMW-PTP and its *fast* and *slow* isoforms on the MDA-MB-435 cell line and knockdowns. Error bars represent standard deviation (n=4). *p<0.05 compared to KD NT and MDA-MB-435. KD NT – scramble sequences siRNA (control); CI, CII; CIII; CV – knockdown of total LMW-PTP; CIV – knockdown of LMW-PTP slow isoform.

Given the controversial role of LMW-PTP in tumor growth and progression, this study aimed at clarifying the importance of LMW-PTP isoforms in breast tumor cell growth, migration and invasion.

Our results show that blocking total LMW-PTP and its *slow* isoform by siRNA in the MDA-MB-435 cell line, a breast cancer invasive cell line, results in increased migratory potential, which our results suggest to occur through RhoA. Therefore, we suggest that the control of LMW-PTP expression, with the consequent balance of RhoA activation, may be a pathway through which the migratory potential of cells is regulated, indicating that LMW-PTP may have an important role in cell migration. There seems to be a differential effect of the two isoforms, with the *fast* isoform having a more important role in cell migration, which may indicate that this isoform is involved in a later stage of tumor development and the *slow* isoform in an earlier stage.

## Methods

### Cell culture

The breast cancer cell line MDA-MB-435 was obtained from the American Type Culture Collection (ATCC number HTB-129) and grown in Dulbecco’s Modified Eagle’s Medium (DMEM, Gibco - Foster City, CA, USA), supplemented with 10% (v/v) fetal bovine serum (FBS), penicillin (100 units/ml) and streptomycin (10 µg/ml), in a humidified atmosphere of 5% CO_2_ at 37°C. All experiments were performed on cells with population doublings between 84 and 100. Knockdown efficiency was confirmed in cells incubated in serum-free DMEM for 48h prior to RNA extraction and assessment of LMW-PTP activity. However, and since for proliferation, migration and invasion assays cells have to be maintained in complete growth medium according to manufacturer’s instructions, all experiments were performed in complete growth medium, in order for the results to be comparable.

### Knockdowns of LMW-PTP isoforms

Five different siRNA sequences were designed to specifically knockdown the total protein (KD LMW-PTP) (GenBank NC_000002.11) (CI#1 5’-GCA AGA CAG ATT ACC AAA GAA-3’; CII#2 5´-GCC TGT TGS GAC TTA GAT AAT-3´; CII#3 5´ CTA TGT ATG GAT GAA AGC AAT3´; CV#5 5´-GAA CTA CTT GGG AGC TAT GAT-3´) and the *slow* isoform (KD LMW-PTP slow) (GenBank accession number NM_007099.3) (CIV#4 5´- GCC CAT AAA GCA AGA CAG ATT-3’). One scramble (non-targeted – KD NT) siRNA was used as control. The lentivirus containing these sequences were a kind gift from Prof. Luis Moita (IMM, Portugal), who designed the sequences and constructed the lentiviral vectors. Given these were part of a pre-existent library that did not include siRNAs for the specific knockdown of the fast isoform, we only used the already existent siRNAs.

Infection was performed 24h after plating 3.5x10^4^ cells per well in 96 well plates, by incubating the cells with the lentiviral vectors for 1h30min at 600*g*, 37°C. Positive knockdowns of MDA-MB-435 were selected with Puromycin after determining the optimal concentration of 0.4 µg/mL. Efficiency of infection was determined by real-time RT-PCR using TaqMan® with primers/probe specific for each of the isoforms, and the Human GAPD Endogenous Control - Applied Biosystems (Foster City, CA, USA).

**Figure 2:**
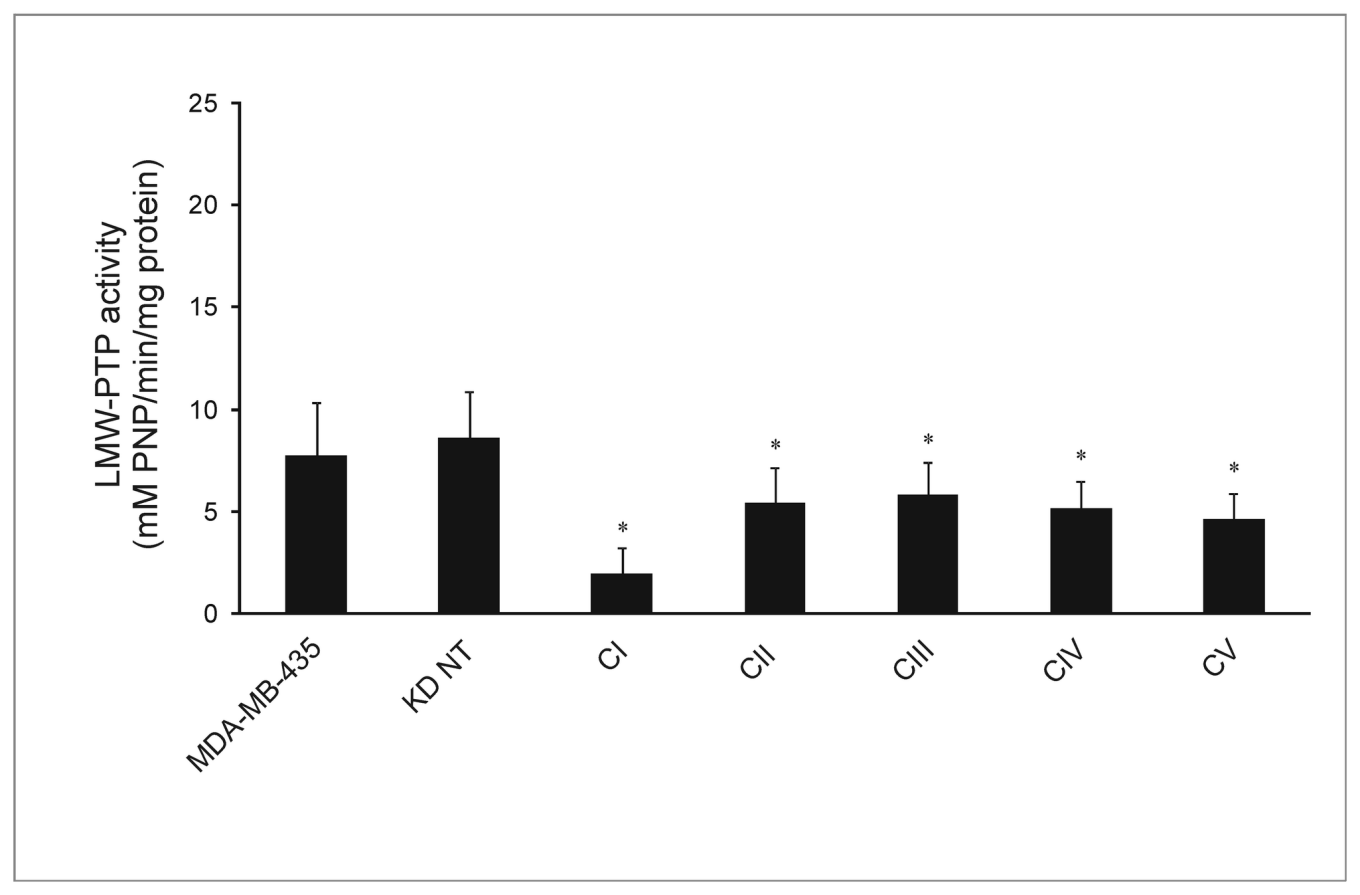
LMW-PTP activity on MDA-MB-435 cell line and knockdowns. Error bars represent standard deviation (n=4). *p<0.05 compared to KD NT and MDA-MB-435. KD NT – scramble sequences siRNA (control); CI, CII; CIII; CV – knockdown of total LMW-PTP; CIV – knockdown of LMW-PTP slow isoform.

### RNA isolation and real-time RT-PCR

Total cellular RNA was isolated with the RNeasy mini kit (Qiagen – Foster City, CA, USA). RNA was quantified by absorbance at 260 nm, and purity was determined by absorbance at 280 and 310 nm (NanoDrop, ThermoScientific – Waltham, MA, USA). RNA (1 µg) was converted into cDNA using the QuantiTect® RT kit (Qiagen- Foster City, CA, USA). An aliquot (20 ng) of the cDNA was then amplified in an ABI Prism 7000 real-time RT-PCR unit using the following TaqMan® Gene Expression Assays (Applied Biosystems – Foster City, CA, USA): acid phosphatase 1, soluble (acp1, Hs00962877 m1), acid phosphatase 1 *fast* isoform, soluble (acp1 *fast* isoform, Hs00964348 g1), acid phosphatase 1 *slow* isoform (acp1 *slow* isoform, Hs00246642 m1). Results were normalized to real-time RT-PCR of GAPDH using the Human GAPD Endogenous Control (4333764F Applied Biosystems) and are expressed using the ΔΔCt method.

### LMW-PTP enzymatic activity

Enzymatic activity of LMW-PTP was measured in MDA-MB-435 cells as previously described [7,8]. Briefly, lysis buffer, containing 10mM *p*-nitrophenyl phosphate and 0.1% Triton X-100 in 0.1 M sodium acetate, 10mM EDTA, pH 5.5, was added to cells for 2h. Samples were alkalinized by adding 1M NaOH and absorbance of *p-*nitrophenol (PNP) was measured at 405 nm. Results are expressed in mM PNP/min and normalized to total protein content, determined by Precision Red^TM^ Advanced Protein Assay Reagent from Cytoskeleton (Denver, CO, USA).

### Proliferation assay

Proliferation rates of both the parental cell line and KDs were determined using PrestoBlue (Invitrogen) assay. 1,650 cells per well were plated in 96 well plates and allowed to adhere for 24h. Proliferation was evaluated after 24h, 72h and 96h by incubation with Presto Blue during 2 hours. Fluorescence was determined with a bottom probe on a fluorescent microplate reader (excitation: 560nm; emission: 590nm, Infinite 200 multimode Reader, Tecan – Mannerdorf, Switzerland). Cell proliferation was confirmed by direct cell counting using a hemacytometer, and the results obtained were not different from the results obtained using PrestoBlue.

### Migration assay

Migratory potential of both the parental cell line and KDs were determined using Platypus Technologies Oris™ (Madison, WI, USA) Cell Migration Assay - Collagen I Coated. Briefly, 25,000 cells per well were plated in 96 well plate provided with the assay and allowed to adhere for 24 hours. At that time, stoppers were removed, allowing cells to migrate freely. Wells from which the stoppers were not removed (t=0) were taken as controls. After 24 hours, stoppers from the control wells were removed and all wells were immediately stained with Calcein-AM (Calbiochem – Darnstat, Germany) (1mg/mL) for 1h at 37°C. Fluorescence was determined with a bottom probe on a fluorescent microplate reader (excitation: 485nm; emission: 528nm, Infinite 200 multimode Reader, Tecan), and pictures of wells were taken with a Zeiss (Jena, Germany) Axiovert 200M – Motorized Widefield Fluorescence Microscope.

**Figure 3:**
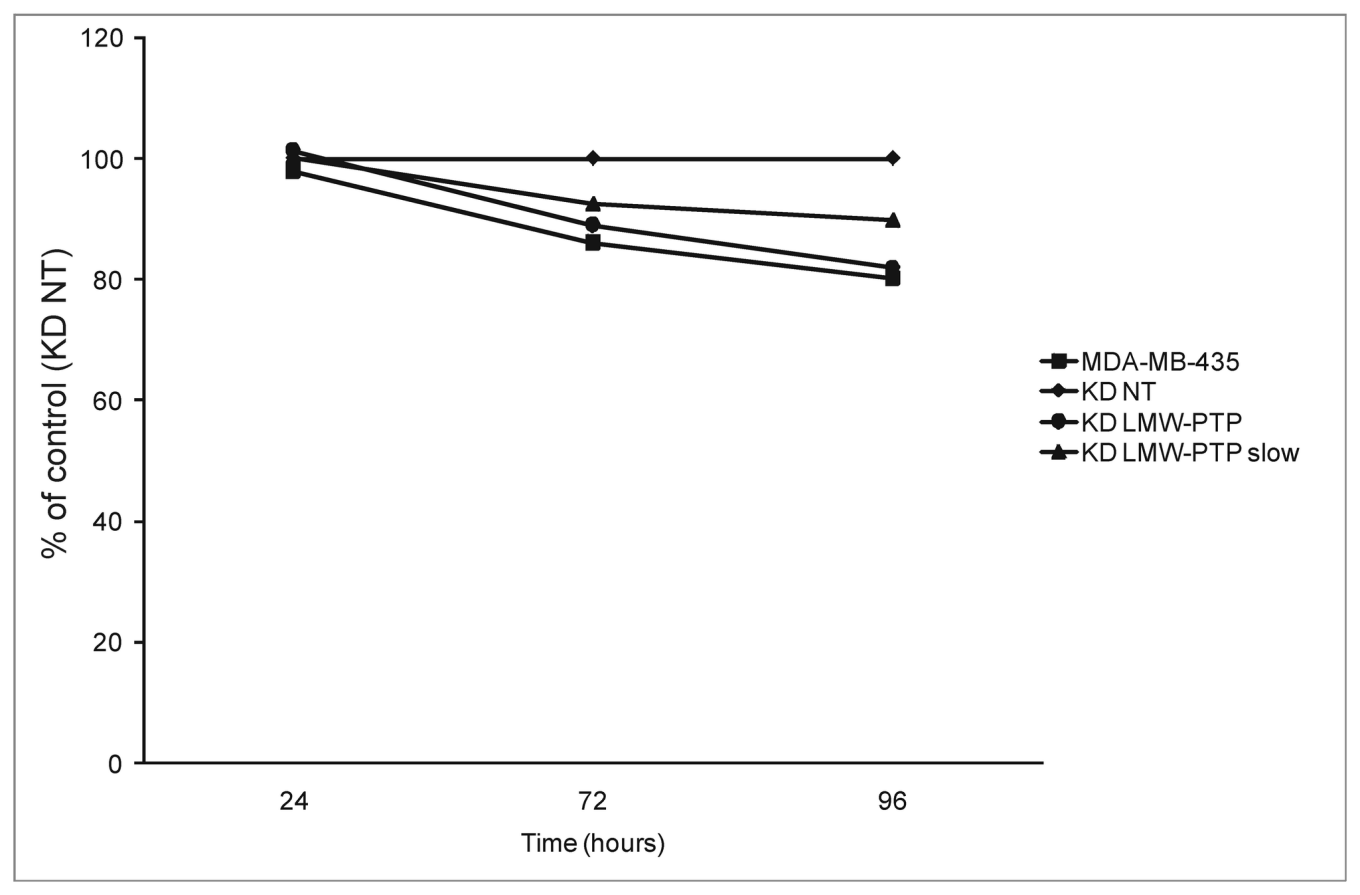
Cell proliferation of different clones compared to KD NT (n=6). p > 0.05 for all comparisons. KD NT – scramble sequences siRNA (control); KD LMW-PTP – total LMW-PTP knockdown; KD LMW-PTP slow – LMW-PTP slow isoform knockdown.

### Invasion Assay

Invasive potential of both the parental cell line and KDs were determined using a 24-well BD BioCoat™ Tumor Invasion System (BD Biosciences – San Jose, CA, USA), following the manufacturer’s instructions. Briefly, 1,650 cells were harvested and plated in the upper chamber, while complete growth medium was added to the lower chamber. The system was incubated for 24 h at 37°C, 5% CO_2_. Cells that invaded and migrated through the BD Matrigel Matrix membrane were post-stained with 4 µg/ml of Calcein-AM (Calbiochem - Darnstat, Germany) in Hank’s buffered salt solution at 37°C, 5% CO_2_ for 1h. Fluorescence was determined with a bottom probe on a fluorescent microplate reader (excitation: 494nm; emission: 517nm, Infinite 200 multimode Reader, Tecan - Mannerdorf, Switzerland).

**Figure 4:**
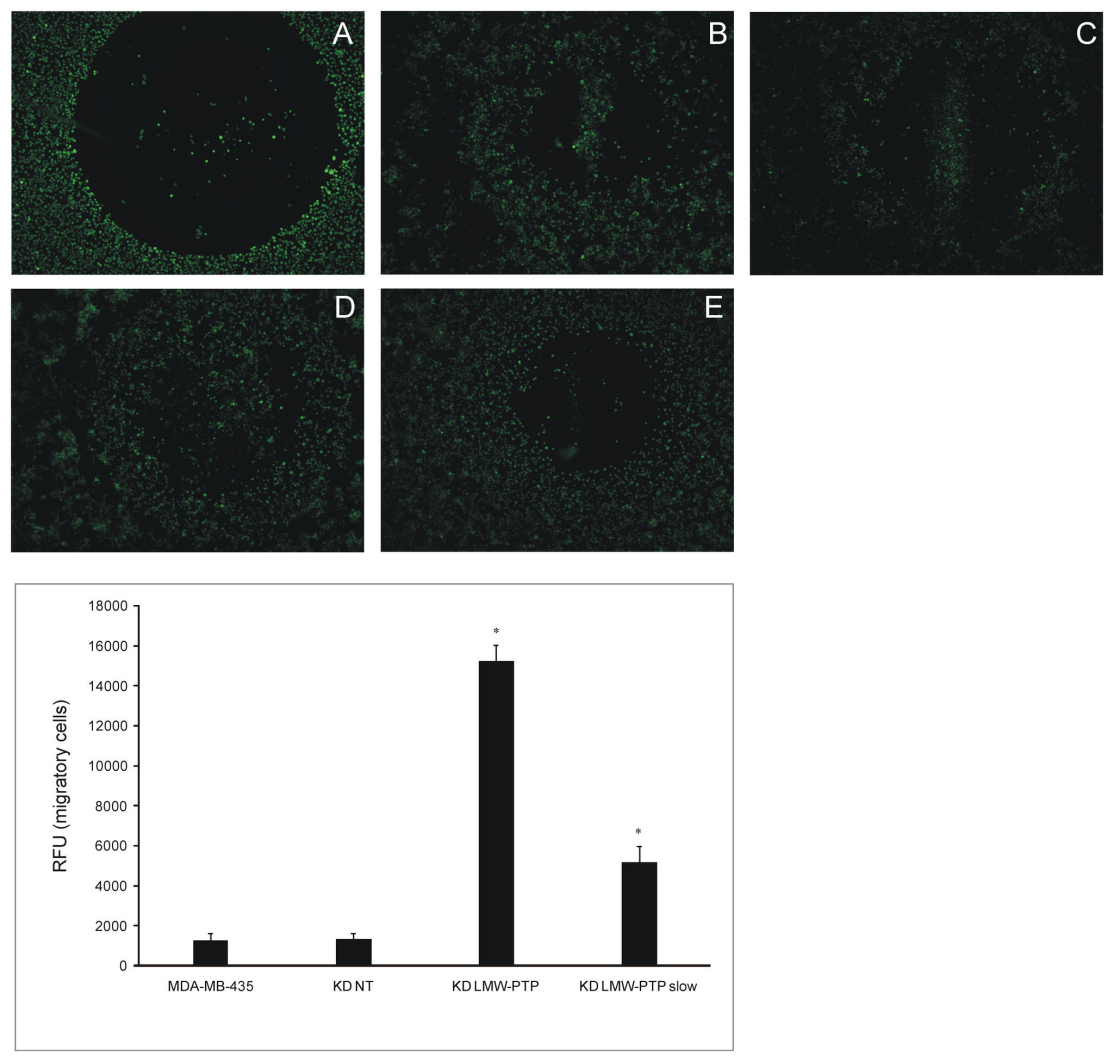
Cell migration of LMW-PTP KDs and MDA-MB-435 compared to KD NT. A-E Representative images of cell migration assay. A: t=0, before migration; B-E: t=24h after migration. B: MDA-MB-435; C:KD NT; D:KD LMW-PTP; E: KD LMW-PTP *slow* isoform. Error bars represent standard deviation (n=3). *p<0.05 compared to KD NT and MDA-MB-435. KD NT – scramble sequences siRNA (control); KD LMW-PTP – total LMW-PTP knockdown; KD LMW-PTP slow – LMW-PTP slow isoform knockdown.

### Rho A activation

RhoA activation of both the parental cell line and KDs were determined using the colorimetric assay RhoA G-LISA™ Activation Assay (Cytoskeleton – Denver, CO, USA). Results were normalized by total protein and not total RhoA protein, according to the manufacturer’s instructions.

**Figure 5:**
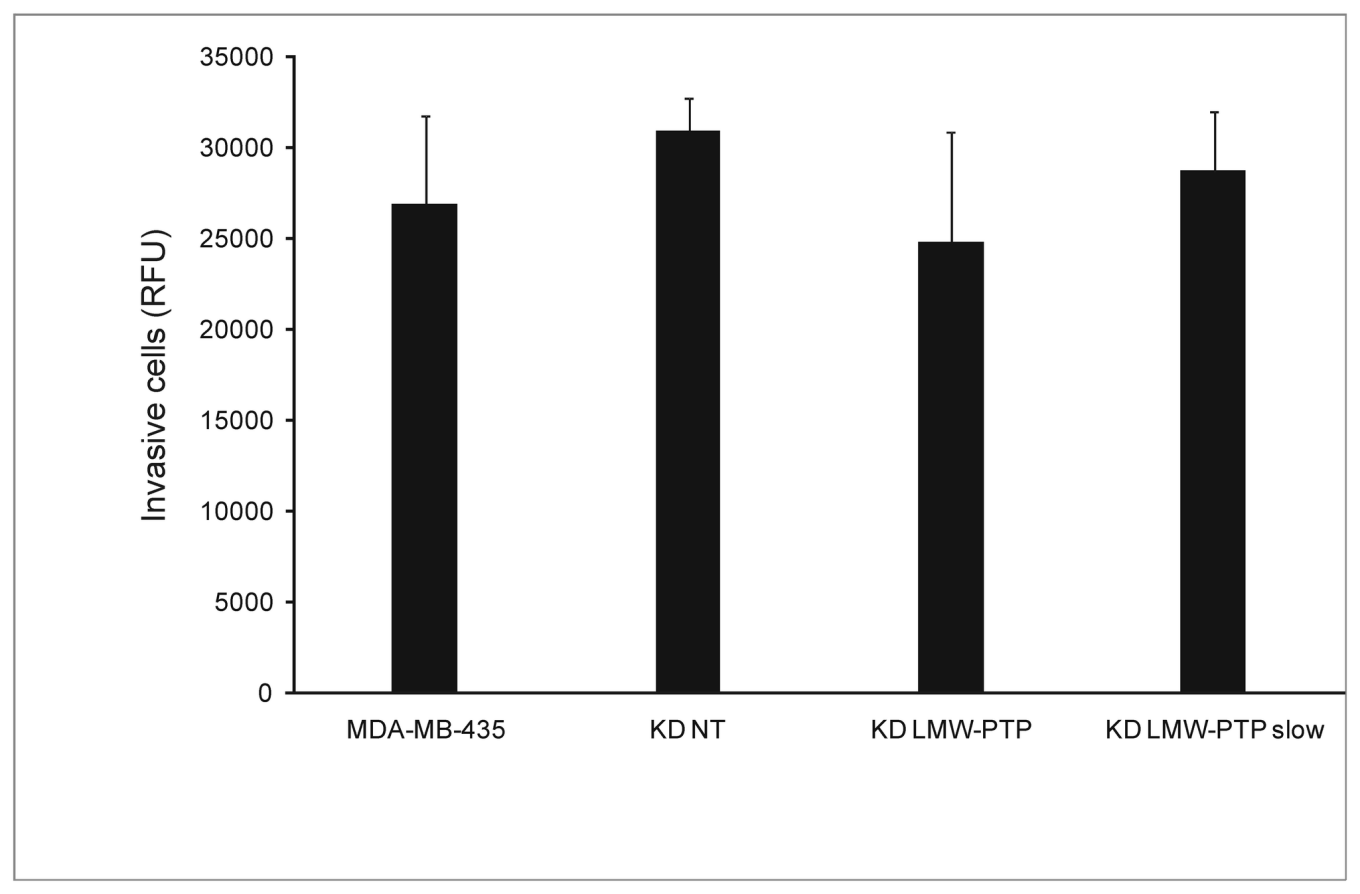
Cell Invasion of LMW-PTP KDs and MDA-MB-435. Error bars represent standard deviation (n=3). p > 0.05 for all comparisons. KD NT – scramble sequences siRNA (control); KD LMW-PTP – total LMW-PTP knockdown; KD LMW-PTP slow – LMW-PTP slow isoform knockdown.

### EphA2 dephosphorylation

Levels of EphA2 dephosphorylation (ratio EphA2 phosphorylated/EphA2 Total) on both the MDA-MB-435 parental cell line and LMW-PTP knockdowns were determined using the ELISA commercial kits Human Total EphA2 DuoSet IC and Human Phospho-EphA2 DuoSet IC (R&D Systems - Minneapolis).

### Statistical analysis

All data are expressed as mean ± standard deviation. Significance was established by Student’s t-test or ANOVA and post-hoc Sidak, as appropriate. Differences were considered significant at p < 0.05.

The study does not need ethical approval.

## Results

### LMW-PTP knockdowns

In order to study the influence of LMW-PTP and its isoforms on the migratory and invasive potential of an epithelial tumor cell line, we produced knockdowns of these proteins using siRNA. After screening 6 tumor cell lines [9] we chose MDA-MB-435 as a model of a breast invasive tumor cell line. This choice was based on the differences of endogenous expression between LMW-PTP fast and slow isoforms [9] and the high invasive potential of this cell line.

Efficiency of knockdowns was confirmed by evaluation of mRNA expression and LMW-PTP enzymatic activity.

Figure 1 shows mRNA expression levels of the parental cell line and all KDs compared to control (KD NT). Knockdown of total LMW-PTP and the two LMW-PTP isoforms was achieved in 3 clones (CII, CIII and CV) and clone CIV was a specific knockdown of the *slow* isoform.

To confirm these results we determined the LMW-PTP enzymatic activity – Figure 2. All five clones showed decreased enzymatic activity compared to KD NT.

Based on these results, we chose two clones for further studies: clone CIV (KD LMW-PTP slow), for being the only clone that showed a specific knockdown of the *slow* isoform, and clone CV (KD LMW-PTP) due to being the clone where the knockdown of LMW-PTP was more effective: 95% compared to 80% of CII and CIII.

**Figure 6:**
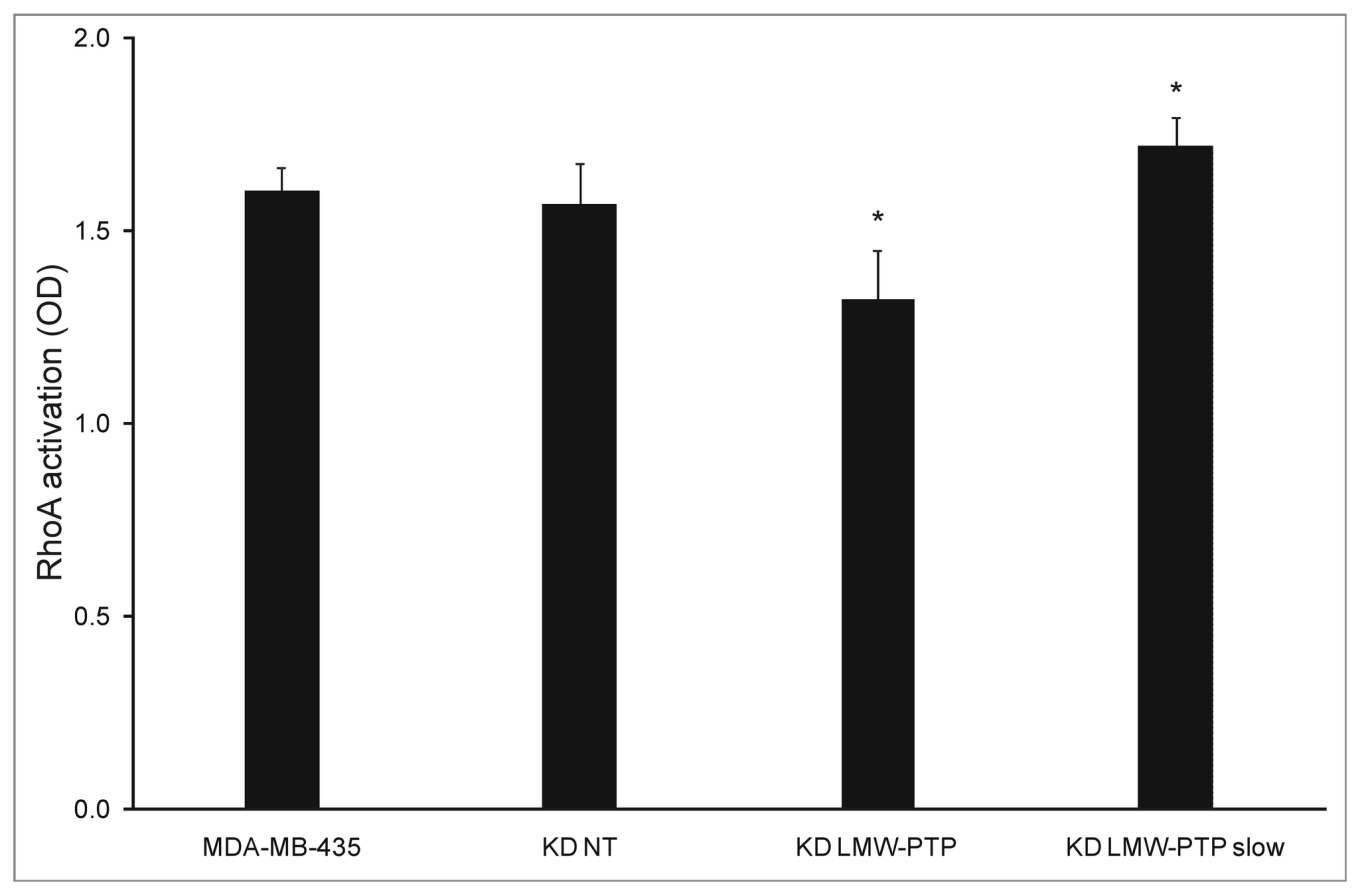
RhoA activation in LMW-PTP KDs and MDA-MB-435. Error bars represent standard deviation (n=3). *p<0.05 compared to KD NT and MDA-MB-435. KD NT – scramble sequences siRNA (control); KD LMW-PTP – total LMW-PTP knockdown; KD LMW-PTP slow – LMW-PTP slow isoform knockdown.

### Proliferation rate is not altered when LMW-PTP is suppressed

Tumor cells are known to have high rates of proliferation, and it has been reported that the LMW-PTP *slow* isoform causes growth arrest [10]. Therefore, we evaluated how the knockdowns of LMW-PTP could interfere with the growth rate of MDA-MB-435. Results show that knockdowns do not change the growth rate of these cells – Figure 3. Cell proliferation was confirmed by direct cell counting using a hemacytometer, and the results obtained were not different from the results obtained using PrestoBlue.

**Figure 7:**
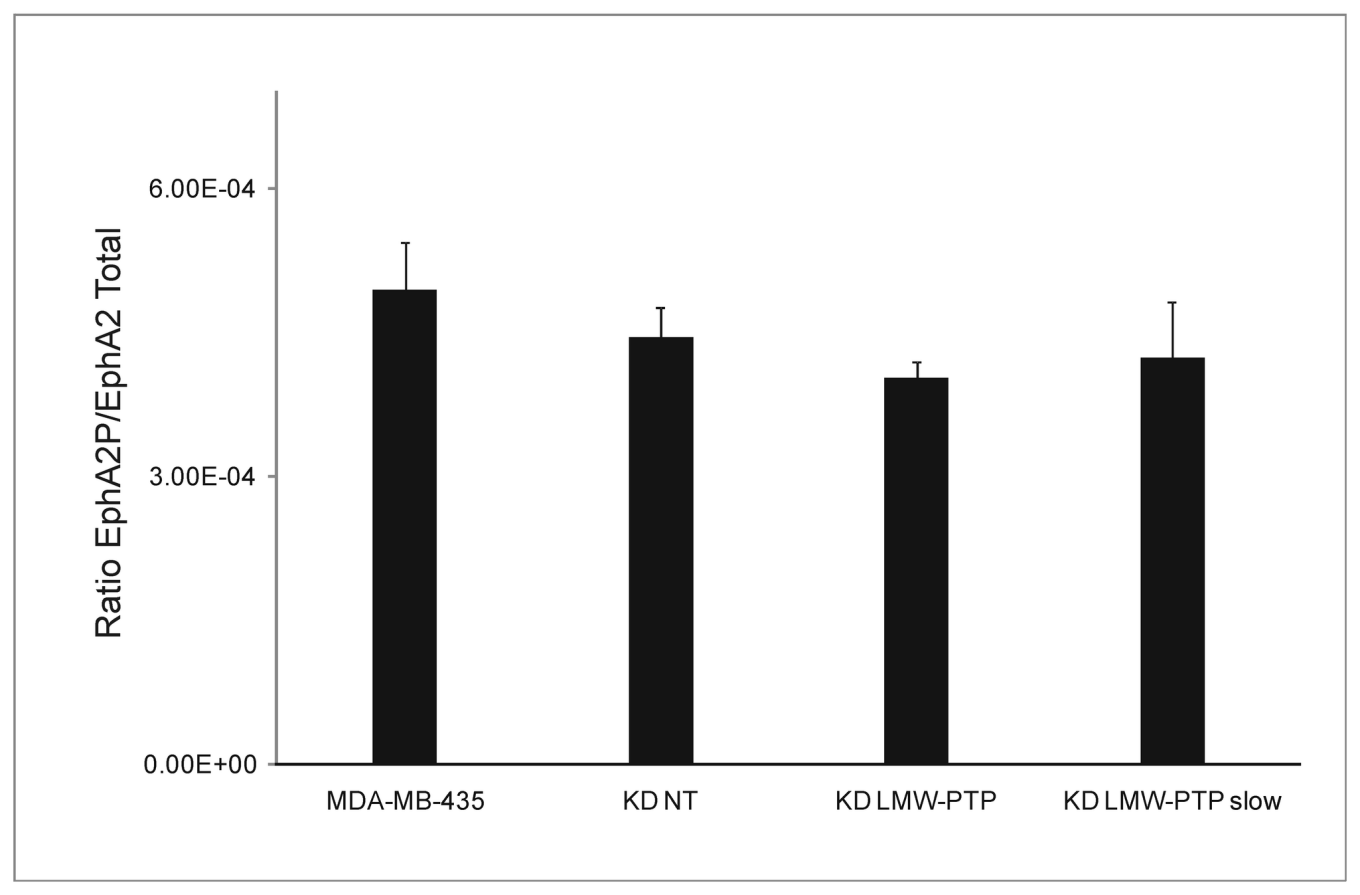
Dephosphorylated EphA2 in LMW-PTP KDs and MDA-MB-435. Error bars represent standard deviation (n=3). p > 0.05 for all comparisons. KD NT – scramble sequences siRNA (control); KD LMW-PTP – total LMW-PTP knockdown; KD LMW-PTP slow – LMW-PTP slow isoform knockdown.

### Suppression of LMW-PTP induces MDA-MB-435 migration

Migratory potential is important for cancer cells to spread and colonize different organs, thereby initiating the metastatic process. We evaluated if the LMW-PTP knockdowns changed the migratory potential of this cells. Both KD LMW-PTP *slow* and KD LMW-PTP migrate more than KD NT and the parental cell (Figure 4)

### Suppression of LMW-PTP does not change MDA-MB-435 invasion potential.

To determine how the suppression of LMW-PTP could influence the invasive potential of MDA-MB-435 cells, we also evaluated the invasive potential of these clones. Analysis of the invasive potential in a Matrigel matrix showed that there were no differences between any of the KD LMW-PTP and MDA-MB-435 (Figure 5).

### Rho A activation is decreased when LMW-PTP is suppressed

To further understand the mechanism by which LMW-PTP can affect the migratory potential, we evaluated the activation status of RhoA. RhoA is an important molecule that regulates cellular adhesion and migration. Results show that RhoA activation is altered in the two knockdowns (Figure 6): KD LMW-PTP had a decreased RhoA activation, whilst KD LMW-PTP *slow* had an increased RhoA activation.

### Phosphorylation status of EphA2 is not altered when LMW-PTP is suppressed

Given the EphA2 receptor can be a substrate for LMW-PTP, we determined if knockdowns of LMW-PTP influenced the phosphorylation levels of this receptor. Analysis of Figure 7 shows that when LMW-PTP is suppressed the phosphorylation status of EphA2 is not changed; neither on the KD LMW-PTP nor on the KD LMW-PTP *slow*, suggesting that, in this model, LMW-PTP does not influence EphA2 phosphorylation status.

## Discussion

The role of LMW-PTP in tumorigenesis has been controversial. As a phosphatase, it is associated with growth arrest through blocking of signal transduction elicited by kinases, thus being considered an oncosuppressor; however, it has been found to be overexpressed in different types of tumors [11] and associated with poor prognosis [12].

In this study, we examined the effect of LMW-PTP suppression in MDA-MB-435, an aggressive breast cancer cell line. Using 5 siRNAs, we successfully suppressed total LMW-PTP and its *slow* isoform.

Confirmation of LMW-PTP isoforms’ knockdown effectiveness can only be achieved through specific mRNA expression, since protein quantification by western blot analysis is not possible because there are no available antibodies targeting LMW-PTP isoforms. Also, enzymatic activity of the two isoforms cannot be determined separately. However, LMW-PTP activity is differentially associated with its isoforms: it is known that the *slow* isoform contributes the most to LMW-PTP activity, also showing a higher enzymatic activity than the *fast* isoform [13]. Accordingly, and given all KDs showed knockdown of the *slow* isoform, all clones had a lower enzymatic activity than KD NT. The exception is the CI. Cells that were infected with lentivirus containing this target siRNA sequence showed a different behaviour of the isoforms. Due to these inconsistent and unexpected results we did not proceed our studies with these clones.

Enhanced proliferation is one of the main characteristics of tumors. The LMW-PTP *slow* isoform can be involved in cell proliferation via two pathways: PDGF-R and EphA2. The relationship between PDGF-R and LMW-PTP has been shown in the NIH3T3 cell line, but there are no previous evidences of this interaction in tumor cell lines. In NIH3T3, PGDF-R is a substrate of the LMW-PTP *slow* isoform. Through dephosphorylation of this receptor, the LMW-PTP *slow* isoform blocks PDGF-induced signalling, decreasing cell growth [10]. However, our results show no differences in the proliferation rates of the KDs. We hypothesize that, given MDA-MB-435 has a high proliferation rate, with a population doubling time of 22h, this is probably not under the sole control of PDGF signalling, and hence cannot be changed only by the absence of LMW-PTP.

LMW-PTP is also known to dephosphorylate EphA2, increasing tumor cell growth and differentiation [14], being the control of the tumorigenic potential of LMW-PTP associated with EphA2 phosphorylation status. EphA2 is dephosphorylated in a wide range of cancer cells and this phenomena seems to correlate with malignancy: tumor cell growth, survival, migration and invasion [15]. Our results show that suppression of LMW-PTP did not change the phosphorylation status of EphA2, suggesting that, in MDA-MB-435 cells, LMW-PTP is not the main regulator of EphA2 phosphorylation status. The tumorigenic potential of these cells may be so dependent on EphA2 phosphorylation that LMW-PTP *per se* is not sufficient to revert this phenotype. Also, the absence of differences in EphA2 phosphorylation status, between all KDs and control, may explain the same growth rate of all KDs and the control, further supporting our hypothesis. All studies that associate LMW-PTP with dephosphorylation of EphA2 have been performed in transformed normal cells, such as MCF10A, a mammary epithelial cell line: EphA2 overexpression is sufficient to cause tumorigenesis in MCF10A cells. However, others have shown that a dominant negative of LMW-PTP did not change EphA2 phosphorylation level significantly, suggesting that EphA2 may not be a major substrate of LMW-PTP in MCF10A [6], and this may also be the case in MDA-MB-435.

On the other hand, LMW-PTP can also be regulated by EphA2 [6]. These authors showed that this regulation is not direct, proposing Src as an intermediary between EphA2 and LMW-PTP. The proposed mechanism, in MCF10A cells, is that EphA2 overexpression probably promotes destabilization of the adherens junctions through a signalling pathway of recruitment of Src kinase, enhanced LMW-PTP activity, inhibition of p190RhoGAP and activation of RhoAGTPase [6].

Based on this model, we evaluated the activation levels of RhoAGTPase in the KDs. Our results show that KD LMW-PTP decreased RhoA activation, which should be due to suppression of the *fast* isoform, whilst KD LMW-PTP *slow* activated RhoA. RhoA activation is controlled by GEFs (guanosine nucleotide exchange factors) and GAPs (GTPase activating proteins). One important GAP is p190RhoGAP. This protein is involved in cytoskeleton rearrangement and seems to be one of LMW-PTP cytoskeletal (*fast*) associated fraction specific substrates [3]. Thus, suppression of LMW-PTP *fast* will cause p190RhoGAP activation with the consequent inactivation of RhoA. As for the effect of the *slow* isoform on RhoA, there are no reports concerning this isoform’s ability to interact with p190RhoGAP. We may hypothesize, based in other authors’ results [16,17] that the interaction between the LMW-PTP slow isoform and p190RhoGap may occur through Src kinase: knockdown of the LMW-PTP slow isoform could inactivate Src kinase [16], which will cause p190RhoGAP inactivation and consequently RhoA activation [17].

Our results show that the LMW-PTP *slow* isoform has the opposite role of the *fast* isoform regarding RhoA activation. Regardless of RhoA increased or decreased activation the effect on the migratory potential of cells is the same: both KD LMW-PTP and KD LMW-PTP *slow* cause an increase in the migration potential of MDA-MB-435, with the former having a higher migratory potential (p<0.05). These apparently controversial results may be explained by previous reports showing that regulation of cell-cell adhesion and, consequently, cell migration, can be achieved through the balance between RhoA GTP/RhoA GDP [18]. Therefore, we hypothesize that the larger increased migration potential of the KD LMW-PTP is mainly due to the lack of the *fast* isoform, suggesting that the *fast* isoform may be more important for the metastatic process than the *slow* isoform, since migration is a characteristic of metastatic tumor cells.

After migration, metastatic cells have to be able to invade surrounding tissues to colonize distante organs. Although it is known that LMW-PTP interacts with β-catenin and E-cadherin [19], molecules that regulate epithelial-mesenchymal transition and consequently invasion, LMW-PTP knockdowns had no effect on the invasive potential, leading us to suggest that in MDA-MB-435 cells the invasive potential is not under control of LMW-PTP.

## Conclusions

Taken together, our results show that regulation of LMW-PTP expression and activity, with the consequent balancing of RhoA activation, affects the migratory potential of these cells. This effect is more pronounced in the KD LMW-PTP, suggesting that the *fast* isoform has a more important role in cell migration and can thus be more prominent in tumor progression than in tumor growth, whilst the *slow* isoform may be important on an earlier stage of the tumorigenic process.

The *fast* and *slow* isoforms seem to have opposite roles in RhoA activation, although leading to the same final effect of increased migratory potential. Apparently, both the increase or decrease of RhoA activation will have the same effect on cell migration, suggesting that any deregulation of RhoA, regardless of being activation or inhibition, will affect cell migration. Therefore, regulation of LMW-PTP is an important feature for cancer cell migration.

Finally, we propose that new therapeutic approaches may be considered using not only total LMW-PTP but targeting specifically its two main isoforms, *fast* and *slow*.
